# Cardiovascular mortality risk in young adults with isolated systolic hypertension: findings from population-based MONICA/KORA cohort study

**DOI:** 10.1038/s41371-021-00619-z

**Published:** 2021-10-14

**Authors:** Seryan Atasoy, Martin Middeke, Hamimatunnisa Johar, Annette Peters, Margit Heier, Karl-Heinz Ladwig

**Affiliations:** 1 Department of Psychosomatic Medicine and Psychotherapy, Klinikum Rechts der Isar, Technische Universität München, München, Germany; 2 Department of Psychosomatic Medicine and Psychotherapy, University of Giessen and Marburg, Giessen, Germany; 3 Institute of Epidemiology, Helmholtz Zentrum München, German Research Center for Environmental Health, Neuherberg, Germany; 4 Hypertension Center Munich, a European Society of Hypertension (ESH) Center of Excellence, Munich, Germany; 5 Kora Study Centre, University Hospital of Augsburg, Augsburg, Germany; 6 Deutsches Zentrum für Herz-Kreislauf-Forschung (DZHK), Partnersite Munich, Munich, Germany

**Keywords:** Risk factors, Hypertension

## Abstract

The clinical significance of isolated systolic hypertension in young adults (ISHY) remains a topic of debate due to evidence ISHY could be a spurious condition resulting from exageratted pulse pressure amplification in “young tall men with elastic arteries”. Hence, we aimed to investigate whether ISHY is associated with an increased risk of cardivascular (CVD) mortality in a sample of 5597 young adults (49.8% men, 50.2% women) between 25 and 45 years old from the prospective population-based MONICA/KORA cohort. ISHY was prevalent in 5.2% of the population, affecting mostly men (73.1%), and associated with increased smoking, obesity, and hypercholesterolemia in comparison to participants with normal blood pressure (BP). Within a follow-up period of 25.3 years (SD ± 5.2; 141,768 person–years), 133(2.4%) CVD mortality cases were observed. Participants with ISHY had a hazard ratio (HR) of 1.89(1.01–3.53, *p* < 0.05) times higher risk of CVD mortality than participants with normal BP, even following adjustment for CVD risk factors. However, adjustment for antihypertensive medication (HR 0.46; 0.26–0.81, *p* < 0.001) and increasing height (HR 0.96; 0.93–0.99, *p* < 0.05) revealed independently protective effects against CVD mortality, suggesting that although ISHY is associated with an increased risk of CVD mortality, the protective effects of increasing height or antihypertensive medication should be considered in treatment rationale.

## Introduction

Isolated systolic hypertension (ISH) is currently defined as an elevated systolic blood pressure (SBP) > 140 mmHg, combined with a normal diastolic blood pressure (DBP) < 90 mmHg [1]. Consequentially of large artery stiffening and increase in pulse pressure, ISH is the most prevalent subtype of hypertension in the elderly [2]. However, ISH also affects an estimated 2–8% of the younger indivuals by heterogeneous mechanisms [3, 4]. Nevertheless, whether ISH in the young (ISHY) implies a worse outcome and needs antihypertensive treatment remains debated [5].

The discussion of whether ISHY is clinically significant and needs to be treated stems from two lines of conflicting evidence [6, 7]. The “pro” argumentation indicates that ISHY is associated with concurrent risk factors such as obesity [8], smoking [3], insulin resistance, and metabolic syndrome [9]. The pathophysiological mechanism between ISHY and worsened health is attributed to hyperkinetic circulation [10, 11] leading to premature aortic stiffening, as supported by previous studies showing a predominantly elevated cardiac output, stroke volume and heart rate in participants with ISHY [12, 13]. Nevertheless, up to 2/3rd of participants in the aforementioned studies did not present hyperkinetic circulation, leading way for the heterogeneity associated with ISHY.

The “con” side of the argument indicates that ISHY is a spurious condition that should not be treated because it is more prevalent in tall, non-smoking, and physically active men [14–16]. This line of evidence attributes ISHY to exageratted pulse pressure amplification from central to peripheral arteries due to elastic large vessels that are common in tall and young individuals [6]. In line with this, numerous studies have shown that young men with ISHY do not have higher risk factors in comparison to those with normal blood pressure [6, 17, 18], however, the increase in SBP was confined to the upper limbs and not centrally present in the aorta, nor other arteries [19].

The clinical outcome of ISHY on future cardiovascular disease (CVD) risk also remains unresolved, leading to difficulty with management and prognosis. In line with the pro side of the argument, a recent prospective study has shown that men with ISHY had 28% higher CVD and 23% higher CHD (coronary heart disease) mortality risk than men with optimal BP, whereas women with ISHY had 2 times higher CVD and 55% higher CHD mortality risk over 30 years [17]. However, supporting the con side of the argument, findings from the HARVEST study indicate that an elevated pulse pressure in participants <45 years might actually have a protective role against hypertension, and can largely be attributed to a white-coat effect [18]—an acute stress response to medical environments.

Given the discrepancies in the literature, the present investigation aims to extend the clinical characteristics of ISHY in a population-based cohort, while for the first time, including psychosocial stress conditions. Additionally, to clarify the prognostic value of ISHY and whether it should be treated, the absolute and relative risks of CVD mortality will be examined among the current community-dwelling participants.

## Methods

The study population was taken from three independent cross-sectional surveys including 5974 participants aged between 25 and 45 years old who participated in the Monitoring of Trends and Determinants in Cardiovascular Disease (MONICA)/Cooperative Health Research in the Region of Augsburg (KORA) cohort study conducted in 1984/1985, 1989/1990, and 1994/1995 as part of the multinational World Health Organization (WHO) MONICA project [20]. All procedures contributing to this work comply with the ethical standards of the relevant committees and comply with the Helsinki Declaration of 1975, as revised in 2008 and written consent was obtained from all participants. In the current analysis, missing follow up data (*n* = 119), and missing covariates at baseline (*n* = 265) led to a pooled sample 5590 participants. A dropout analysis revealed that subjects with missing information were older compared to subjects with available information (*p* < 0.001).

### Assessment of hypertension

Adhering to WHO MONICA protocol, blood pressure (BP) was measured on the right arm in a sitting position using a Hawksley random-zero sphygmomanometer, and measurements were taken half an hour after the clinical interview in 3-min intervals. The average readings of the second and third measurement were considered for the analyzes. An “actual hypertension” [21] measure was used based on the measured blood pressure values, independently of the use of antihypertensive. In line with the ESC/ESH Hypertension Guidelines, isolated-systolic hypertension in the young was defined as brachial SBP ≥ 140 mmHg and DBP < 90 mmHg, isolated-diastolic hypertension in the young (IDHY) was defined as SBP < 140 mmHg and DBP ≥ 90 mmHg, and systolic-diastolic hypertension in the young (SDHY) was defined as SBP ≥ 140 mmHg and DBP ≥ 90 mmHg [1].

### Socio-demographic and lifestyle factors

Low educational level was considered as having less than 12 years of schooling. Physical activity was considered as engaging in physical activity on average of ≥1 h/week throughout the year. Smoking was based on current and regular smoking of ≥1 cigarette/day.

### Cardio-metabolic factors

Total cholesterol and high-density lipoprotein cholesterol were measured as mg/dL in serum by enzymatic methods (CHOD-PAP, Boehringer Mannheim, Germany) and dyslipidemia was defined as ratio of total cholesterol to high-density lipoprotein cholesterol ≥ 5.0. Type 2 diabetes was self reported and confirmed by a physician diagnosis. Trained medical staff measured the body weight (kg) and height (cm) of all participants anthropometrically as part of a standardized medical examination. Calibration of measuring instruments was ensured through weekly or daily inspections using standard weights. Body mass index (BMI) was calculated as weight in kilograms divided by height in meters squared and obesity was defined as having a BMI ≥ 30 kg/m². History of parental hypertension was based on participant’s report of either parent with hypertension. Cardiovascular (CVD) conditions were based on a self-reported history of myocardial infarction, heart failure, angina, or stroke.

### Psychosocial risk factors

Depressive symptoms were assessed using the sex-specific top tertiles of the depression and exhaustion subscale of the von Zerssen symptom checklist [22]. Living arrangement was assessed by whether the individual lives alone, irrespectively of relationship status. Sleep complaints were adopted from the Uppsala Sleep Inventory [23] and participants were considered to have sleep complaints if they often had difficulty with initiating and/or maintaining sleep. Job strain was assessed in employed participants by the job content questionnaire [24] and was defined by the quadrant approach where continuous job demands and job control variables were dichotomized by median split [25]. Type A traits were assessed by the Framingham Type A Scale with cut-off points of 0.34 for men and 0.33 for women [26].

*Follow-up and mortality endpoints* Death certificates were obtained from local health departments and coded for the underlying cause of death by trained personnel using the 9th revision of the International Classification of Diseases (ICD-9). In the present study, fatal CVD events (ICD-9: 390–459) were used as endpoints. During a mean follow-up period of 25.3-years (SD ± 5.89 years; max: 32 years; 141,768 person years), 133 fatal CVD events (88 in men; 45 in women) were observed. For mortality analyses, event times were calculated as time to death. Subjects without events or with loss to follow-up were censored at the time point of the last follow-up.

### Statistical analysis

The baseline characteristics of the study population between 25 and 45 years with normal BP, ISHY, IDHY, and SDHY were assessed using Pearson’s *χ*^2^ for categorical and analysis of variance for continuous variables, and significance of group differences were tested using multivariate logistic regression and Tukey’s Method in reference to participants with normal BP.

Absolute CVD mortality rates were calculated using Poisson regression adjusted for baseline survey. The relative risk of CVD mortality in participants with ISHY was given as hazard ratios (HR) using Cox proportional hazards models, where normal BP was the reference group. Four multivariate Cox regression models adjusted for [1] age, sex, survey [2] life-style factors [3] somatic risk factors, and history of CVD and [5] continuously increasing height and antihypertensive medication use were included, where the fully adjusted model included all these covariates.

Proportional hazards could be estimated by fitting models stratified by the risk factor categories and plotting the log-log survival curves for each risk factor, which were assessed for parallelism by visual inspection. As severe deviations from parallelism were not observed for any covariates of CVD events, proportional hazards were assumed. To ensure power of the analyses was at least 80%, a log-rank test was conducted for comparison of survival rates of CVD mortality in the BP categories.

For main analyses, a *p* value < 0.05 was statistically significant. All statistical evaluations were performed using SAS 9.4. The analysis and the description in this manuscript follow the STROBE guidelines for cohort studies.

## Results

The present data were derived from a total of 5597 participants (49.8% men, 50.2% women) at baseline with a mean age of 35.2 years (SD ± 6.1), of whom 5.2% (73.1% men, 26.9% women) had ISHY, 6.7% (71% men, 29% women) had IDHY and 7.6% (73.1% men, 26.9% women) had SDHY. At baseline, men with ISHY were taller than participants with IDHY and SDHY, yet there was no significant difference of height between participants with ISHY and normal BP. However, women with ISHY were more likely to have a shorter height than all remaining groups.

As shown in Table 1, ISHY had increased concurrent risk factors in comparison to participants with normal BP, whereby they were more likely to be male, smoke regularly, have obesity, dyslipidemia, and high pulse pressure. On the other hand, participants with ISHY and normal BP had no significant difference in age and physical activity, whereas participants with ISHY had even lower levels of job strain and type A traits than participants with normal BP.

**Table 1:** Baseline characteristics of CVD risk factors in the MONICA/KORA Cohort, according to BP groups (*N* = 5597).

Baseline characteristics	Prevalence, *n* (%)				
	Total	Normal BP	ISHY	IDHY	SDHY
Total	5597 (100.0)	4504 (80.4)	290 (5.2)	376 (6.7)	427 (7.6)
Men	2790 (49.8)	1999 (44.4)	212 (73.1)^***^	267 (71.0)^***^	312 (73.1)^***^
Women	2807 (50.2)	2505 (55.6)	78 (26.9)^***^	109 (29.0)^***^	115 (26.9)^***^
Mean age (years) (±SD)	35.2 (6.1)	34.7 (6.1)	35.5 (6.8)	37.4 (5.4)^***^	38.6 (5.3)^***^
Education (≤12 yrs)	3647 (65.2)	2907 (64.5)	192 (66.2)	246 (65.4)	302 (70.7)
Smoking	2052 (36.7)	1640 (36.4)	127 (43.8)^*^	126 (33.5)	159 (37.2)
Physically inactive	2791 (49.9)	2208 (49.0)	146 (50.3)	206 (54.8)^*^	231 (54.1)^*^
Systolic BP (±SD)	123.8 (14.5)	119.0 (10.4)	146.0 (6.4)^***^	132.4 (5.3)^***^	151.9 (11.8)^***^
Diastolic BP (±SD)	78.0 (11.1)	74.5 (8.4)	81.6 (7.3)^***^	93.2 (3.2)^***^	98.9 (7.5)^***^
High pulse pressure	481 (8.6)	183 (4.1)	158 (55.0)^***^	0	65 (15.2)^***^
Height (cm) (±SD) *Men*	176.7 (6.6)	176.9 (6.5)	177.2 (6.9)	175.4 (6.4)^*^	175.6 (6.5)^*^
*Women*	163.3 (5.9)	163.4 (5.6)	161.5 (5.8)^*^	162.7 (5.9)	162.7 (6.1)
Weight (kg) (±SD)	73.2 (14.5)	71.1 (13.5)	79.6 (15.0)^***^	80.2 (15.1)^***^	84.2 (15.8)^***^
BMI	25.2 (4.1)	24.7 (3.7)	26.6 (4.5)^***^	27.1 (4.5)^***^	28.3 (4.7)^***^
Obesity^a^	636 (11.4)	384 (8.5)	43 (14.8)^***^	89 (23.7)^***^	120 (28.1)^***^
Dyslipidemia^b^	1329 (23.7)	889 (19.7)	94 (32.4)^***^	150 (39.9)^***^	196 (45.9)^***^
Type 2 diabetes	29 (0.52)	17 (0.38)	3 (1.03)	3 (0.80)	6 (1.41)^**^
Parental history	2249 (40.2)	1749 (38.9)	128 (44.1)	164 (43.6)	208 (48.7)^***^
Treatment^c^	158 (2.8)	70 (1.6)	17 (5.9)^***^	17 (4.5)^***^	54 (12.7)^***^
History of CVD^d^	97 (1.73)	73 (1.6)	3 (1.03)	11 (2.9)	10 (2.3)
Depressive symptoms	993 (19.2)	800 (19.2)	44 (16.9)	75 (21.7)	74 (18.9)
Job strain	1035 (26.3)	836 (27.0)	40 (17.6)^**^	64 (22.1)^*^	95 (29.2)
Type A traits	2422 (45.6)	1957 (45.7)	102 (37.8)^*^	187 (52.1)^*^	176 (43.9)
Living alone	1218 (21.8)	997 (22.1)	64 (22.1)	76 (20.2)	81 (19.0)

*BP* blood pressure, *BMI* body mass index, *CVD* cardiovascular disease. *p < 0.05, **p < 0.01, ***p < 0.001.

^a^Obesity: BMI > 30.

^b^Ratio of total cholesterol to high-density lipoprotein cholesterol ≥ 5.0.

^c^Antihypertensive medication.

^d^History of myocardial infarction, heart failure, angina, or stroke.

In comparison to participants with normal BP, IDHY and SDHY posed a more amplified risk factor profile than ISHY, whereby they were more likely to be older, physically inactive, have obesity, dyslipidemia, history of CVD conditions, type 2 diabetes. An exception to this trend was with smoking, which remained substantially higher in participants with ISHY.

### Absolute CVD mortality rates

**Fig. 1:**
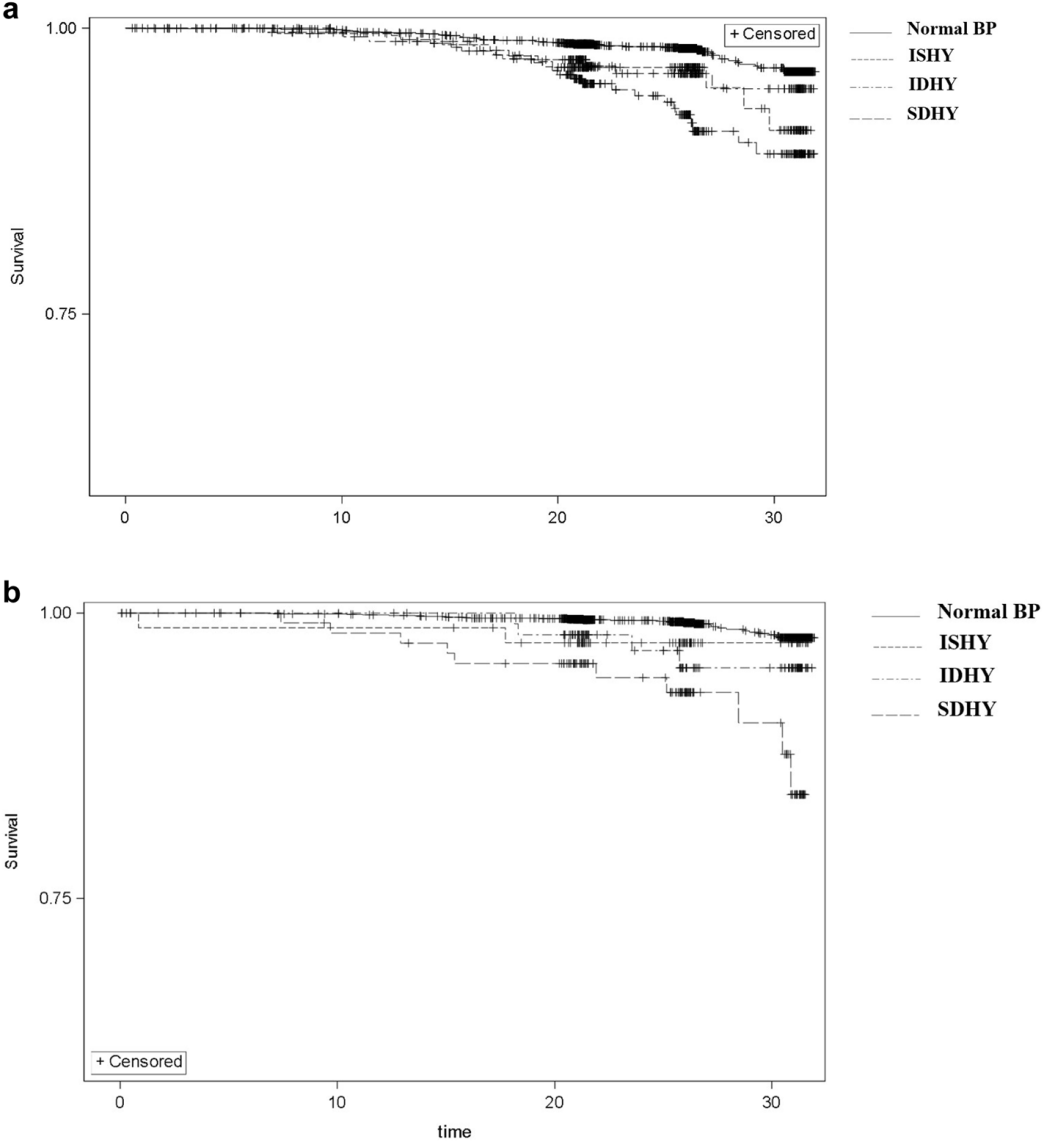
**a** Kaplan-Meier survival curve for CVD mortality in men according to blood pressure groups in the MONICA/KORA Cohort (*n* = 2790). *Analyses based on 88 fatal CVD events. *BP* blood prssure; *CVD* cardiovascular disease; *ISHY* isolated systolic hypertension in the young; *IDHY*  isolated diastolic hypertension in the young; *SDHY*  systolic-diastolic hypertension in the young. **b** Kaplan-Meier survival curve for CVD mortality in women according according to blood pressure groups in the MONICA/KORA Cohort (*n* = 2807). *Analyses based on 45  fatal CVD events. *BP* blood pressure; *CVD*  cardiovascular disease; *ISHY* isolated systolic hypertension in the young; isolated diastolic hypertension in the young; *SDHY* systolic-diastolic hypertension in the young.

**Fig. 2:**
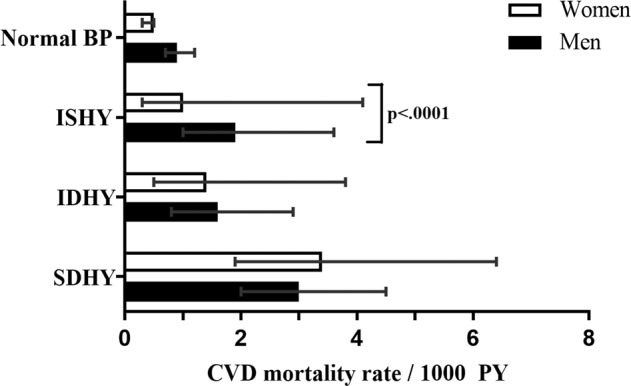
Sex-stratified absolute CVD mortality rates of the MONICA/KORA Cohort, according to blood pressure groups (*N* = 5597). *Analyses based on 133 fatal CVD events **Error bars represent 95% CI. *BP* blood pressure; *CVD * cardiovascular disease; *ISHY*  isolated systolic hypertension in the young; *IDHY*  isolated diastolic hypertension in the young; *SDHY*  systolic-diastolic hypertension in the young.

During a mean follow-up period of 25.3 years (SD ± 5.89), 133 (2.4%) fatal CVD events were observed, including 88 events in men and 45 events in women  (Fig. 1). In general, participants with ISHY had a significantly higher absolute CVD mortality rate than participants with normal BP (1.5 vs. 0.6 cases/1000 person–years (py), *p* = 0.001). Likewise, participants with IDHY (1.3 cases/1000 py, *p* = 0.004) and SDHY (2.6 cases/1000 py, *p* < 0.0001) experienced higher CVD mortality in comparison to participants with normal BP. However, as shown in Fig. 2, the mortality rate revealed significant sex differences, whereby men with ISHY were nearly two times more likely to experience CVD mortality than women with ISHY (*p* > 0.0001). However, large sex differences were not observed in participants with IDHY or SDHY.

### Relative risk of CVD mortality

Within a mean of 25.3 years of follow-up, participants with ISHY had a 1.89 (95% CI 1.01–3.53, *p* < 0.05) times higher relative risk of CVD mortality in comparison to participants with normal BP (Table 2). Participants with SHDY had an even higher risk of CVD mortality by a hazard ratio of 2.36 (95% CI 1.50–3.72, *p* < 0.001), but participants with IDHY did not experience a significantly higher risk of CVD mortality than participants with normal BP (HR 1.44; 95% CI 0.80–2.59, *p* = 0.28). Altogether, ISHY was associated with a lower risk of CVD mortality than participants with SDHY (HR 0.76; 0.39–1.49, *p* = 0.43), but a higher risk than participants with IDHY (HR 1.32; 95% CI 0.61–2.88, *p* = 0.47), although these differences did not reach statistical significance.

**Table 2:** Hazard ratios of CVD mortality in participants with ISHY (*n* = 290), IDHY (*n* = 376), and SDHY (*n* = 427) in comparison to normal BP participants (*n* = 4504) from the MONICA/KORA Cohort (HR, 95% CI, *p*).

Variable	Model 1	Model 2	Model 3	Model 4
	*HR (95% CI)*	*HR (95% CI)*	*HR (95% CI)*	*HR (95% CI)*
ISHY	2.19 (1.18–4.06)*	2.13 (1.15–3.95)^*^	2.01 (1.08–3.74)^*^	1.89 (1.01–3.53)*
IDHY	1.67 (0.93–2.97)	1.69 (0.94–3.02)	1.52 (0.85–2.72)	1.44 (0.80–2.59)
SDHY	3.10 (2.02–4.77)^***^	3.09 (2.00–4.76)^***^	2.61 (1.67–4.07)^***^	2.36 (1.50–3.72)^**^
Men	1.85 (1.27–2.69)^**^	1.73 (1.18–2.52)^**^	1.38 (0.92–2.08)	2.02 (1.19–3.44)^**^
Age (years)	1.10 (1.06–1.13)^***^	1.10 (1.07–1.14)^***^	1.09 (1.06–1.13)^***^	1.08 (1.05–1.12) ^***^
Low education^a^	1.49 (1.00–2.21)^*^	1.32 (0.89–1.97)	1.22 (0.82–1.83)	1.18 (0.79–1.77)
Smoking	-	1.86 (1.32–2.65)^**^	1.78 (1.25–2.53)^**^	1.83 (1.28–2.60)^**^
Physical inactivity	-	1.50 (1.05–2.15)^*^	1.39 (0.97–2.00)	1.34 (0.93–1.93)
Obesity	-	-	1.43 (0.94–2.20)	1.36 (0.89–2.09)
Dyslipidemia^b^	-	-	1.82 (1.23–2.67)^**^	1.73 (1.18–2.55)^**^
History of CVD^c^	-	-	2.40 (1.11–5.20)^*^	2.18 (1.01–4.75)^*^
Type 2 diabetes	-	-	4.81 (1.90–12.16)^**^	4.72 (1.88–11.87)**
Treatment^d^	-	-	-	0.46 (0.26–0.81)^**^
Height (cm)	-	-	-	0.97 (0.95–0.99)^*^

Analyses based on 133 fatal CVD events.

*BP* blood pressure, *CVD* cardiovascular disease, *ISHY* (isolated systolic hypertension in young adults

*p < 0.05, **p < 0.01, ***p < 0.001.

^a^≤12 years of education.

^b^Ratio of total cholesterol to high-density lipoprotein cholesterol ≥ 5.0.

^c^History of myocardial infarction, heart failure, angina, or stroke.

^d^Use of antihypertensive medication.

Concurrent risk factors, including being male (HR 2.98; CI 1.50–5.86, *p* = 0.002), smoking (HR 1.83; 95% CI 1.28–2.60, *p* < 0.001) dyslipidemia (HR 1.85; 95 % CI 1.12-3.07, *p* = 0.02), type 2 diabetes (HR 4.72; CI 1.88–11.87, *p* < 0.001) and suffering from a history of CVD conditions (HR 1.78; 95% CI 1.00–3.18, *p* < 0.05) were significant in increasing the risk of CVD mortality in participants with hypertension. On the other hand, protective effects of increasing height and antihypertensive treatment were observed, whereby every 1 cm increase in height was associated with a 4% (HR 0.96; 95% CI 0.93–0.99, *p* = 0.02) lowered risk of CVD mortality, and/or the use of antihypertensive treatment was associated with a 54% (HR 0.46; 95% CI 0.26–0.81, *p* < 0.001) lowered risk of CVD mortality.

Furthermore, sex stratified results in the adjusted models revealed that men with ISHY had a 1.99 (95% CI 0.99–3.99, *p* = 0.05) times higher risk of CVD mortality than men with normal BP, whereas women with ISHY did not experience a significantly higher risk of mortality than women with normal BP (HR 1.29; 95% CI 0.30–5.55, *p* = 0.73).

### Sensitivity analysis

Following the exclusion of participants with CVD conditions and/or antihypertensive treatment (*n* = 238), ISHY remained associated with a 1.94 (95% CI 0.99–3.80, *p* = 0.05) increased risk of CVD mortality, albeit with a borderline statistical significance. Additionally, participants with SDHY experienced a significantly higher risk of CVD mortality in comparison to participants with normal BP (HR 2.35; 95% CI 1.42–3.90, *p* < 0.001), whereas participants with IDHY did not reach a significance in their risk of CVD mortality (HR 1.41; 95% CI 0.73–2.71, *p* = 0.30).

## Discussion

Due to the ongoing dispute about the clinical significance of ISHY [5], the present study focused on the characteristics and prospective risk of CVD mortality associated with ISHY in a population-based cohort. The findings herein confirmed that ISHY is a clinically heterogenous condition [4], whereby participants with ISHY were more likely to be obese, smoke, have increased pulse pressure and somatic risk factors in comparison to participants with normal BP. They were nevertheless of similar age, had comparable physical activity levels, and had an even lower prevalence of psychosocial impairment. Despite the heterogeneity of the condition, participants—with ISHY- largely comprised of men—suffered from 89% higher risk of CVD mortality in comparison to participants with normal BP participants during the follow-up period.

Although previous evidence suggests that ISHY has different underlying mechanisms than ISH in the elderly [5], the baseline characteristics reported herein revealed that risk factors associated with ISHY are generally comparable to older participants, albeit less prevalent. The results herein strengthened previous findings that risk factors including smoking, low education, and high BMI are associated with ISHY [3, 27, 28]. However, the current results concurrently supported previous findings that ISHY is more common in taller men who are more likely to be physically active in comparison to other young hypertension groups [14, 15], but in contrast to this line of evidence, ISHY was associated with smoking more than any other BP group. Hence, the detrimental effect of smoking on arterial stiffness and acceleration of age-related decline in blood pressure amplification in young individuals is thought to be one of the main pathways to ISHY in the current investigation [29, 30].

Despite previously reported associations between ISHY and white coat hypertension [18]—which is appraised as an acute stress response condition—the current study revealed that ISHY was in fact not associated with sustained stress conditions. In contrast, ISHY experienced the lowest job strain and type A behavior pattern in comparison to all remaining BP groups. However, the current findings may be discerned in light of findings linking higher systolic BP to lower psychological distress [31]. Additionally, the lower psychosocial stress conditions in ISHY could be driven by the substantially high smoking rate in these individuals, previously put forth as *Nesbitt´s paradox*. Within this concept, nicotine acts as a physiological stimulant which is actually perceived as tranquilizing to the individual smoking [32] all the while contributing to increased SBP [29].

During a mean 25-years follow up period, ISHY suffered from 1.5 cases/1000 person–years of absolute CVD mortality rates, corresponding to a 1.89 times higher adjusted relative CVD mortality risk in comparison to participants with normal BP. In line with a previous study by Yano et al. [17], “ISH risks were higher than in those with high-normal BP or isolated diastolic hypertension and less than those associated with systolic diastolic hypertension”. However, the overall risk associated with ISHY reported herein - particularly in men - was higher than the ISHY risk reported by Yano et al. [17], whereby ISHY increased incidence of CVD mortality by HR of 1.23 in men and 1.55 in women. However, differences between the two populations, including the larger sample size, more women with ISHY and younger age range of participants between 18 and 49 years may partially explain these discrepancies. Furthermore, it is possible that the results by Yano et al. [17], may have been underestimated as their BP measurement was based on a single casual supine measurement, a position which can result to a lower systolic and higher diastolic BP measurement in comparison to the sitting position [33].

The prospective findings herein further supported attributes of both the “pro” and “con” sides of the ISHY debate [6, 7] – protective effects of increasing height, as well as anti-hypertensive treatment against the risk of CVD mortality, was evident. In line with the aforementioned ‘tall men with elastic arteries’ argument [13–15], each 1-cm increase in height independently decreased the CVD mortality risk by 4%, confirming findings by a recent Mendelian Randomization study showing that genetically predicted height is causally associated with higher systolic BP, as was the case in men with ISHY in the current study, as well as lower arterial stiffness [34]. However, the overall effect of ISHY on the risk of CVD mortality remained substantial even following adjustments for protective factors, indicating that ISHY is an independent and significant predictor of CVD mortality.

The main limitation of the current study is that direct cause and effect relationships cannot be discerned due to lack of ambulatory or central BP measures, white-coat effect estimation with any out-of-office measurement and arterial stiffness parameters. Furthermore, the absence of information about time-varying phenomena during the follow-up period, including the incidence of SDHY in ISDHY and IDHY subjects, changes in BP and/or start of antihypertensive medication or other cardio-protective drug agents is a limitation that is innate in such study designs. Additionally, the mortality cases during the follow-up period were relatively low, however, this study is representative of the real-world situation in which young adults in the general population have increasing lifespans. Similarly, as 73% of baseline ISHY cases were men, the current ISHY-CVD mortality link is largely representative of men rather then women. On the other hand, the strengths of the current study include the reliable measurement of BP, heterogeneity of many participants randomly drawn from the population, as well as the considerable data on psychosocial risk factors.

In conclusion, the difference between baseline SBP and DBP in ISHY is associated with a variety of heterogeneous demographic, lifestyle, anthropometric, somatic, and psychosocial characteristics. Nevertheless, ISHY independently heightens the future risk of CVD mortality – particularly in men. However, as height emerges as an additional factor with a protective role against the risk of CVD mortality, central BP measures should nowadays be monitored for the treatment rationale of tall men who present ISHY.

### Summary Table

#### What is known about the topic

- Previous studies have shown that isolated systolic hypertension in young adults is a clinically heterogenous condition—although isolated systolic hypertension in young adults has been associated with concurrent risk factors including smoking and obesity, it has also been associated with non-clinical factors including ‘elastic arteries’ present in tall and physically active men.
- However, whether isolated systolic hypertension in young adults increases the future risk of cardiovascular mortality remains unresolved—particularly among tall individuals who may have increased systolic blood pressure in the peripheries, yet not centrally.

#### What this study adds

- The study shows that young adults with isolated systolic hypertension have substantially higher concurrent risk factors than normal BP young adults, yet they are also significantly more likely to be taller.
- Young adults with isolated systolic hypertension have a substantially higher risk of cardiovascular mortality in comparison to their normal BP counterparts.
- However, protective effects of antihypertensive medication and increasing height in young adults with systolic hypertension should be considered in treatment rationale.

## Data Availability

The current data and code can be requested from the MONICA/KORA-Myocardial Infarction Registry in Augsburg, Germany.
