# Correction to: Cardiovascular mortality risk in young adults with isolated systolic hypertension: findings from population-based MONICA/KORA cohort study

**DOI:** 10.1038/s41371-023-00804-2

**Published:** 2023-01-23

**Authors:** Seryan Atasoy, Martin Middeke, Hamimatunnisa Johar, Annette Peters, Margit Heier, Karl-Heinz Ladwig

**Affiliations:** 1 Department of Psychosomatic Medicine and Psychotherapy, Klinikum Rechts der Isar, Technische Universität München, München, Germany; 2 Department of Psychosomatic Medicine and Psychotherapy, University of Giessen and Marburg, Giessen, Germany; 3 Institute of Epidemiology, Helmholtz Zentrum München, German Research Center for Environmental Health, Neuherberg, Germany; 4 Hypertension Center Munich, A European Society of Hypertension (ESH) Center of Excellence, Munich, Germany; 5 Kora Study Centre, University Hospital of Augsburg, Augsburg, Germany; 6 Deutsches Zentrum für Herz-Kreislauf-Forschung (DZHK), Partner Site Munich, Munich, Germany

**Keywords:** Risk factors, Hypertension

Correction to: *Journal of Human Hypertension* 10.1038/s41371-021-00619-z, published online 14 October 2021

The original version of this article contained an error. The first and last names of the authors were interchanged. The original article has been corrected.

